# Editor’s note to: A cannabis oracle? Delphi method not a substitute for randomized controlled trials of cannabinoids as therapeutics

**DOI:** 10.1186/s42238-021-00086-w

**Published:** 2021-07-02

**Authors:** David A. Gorelick

**Affiliations:** University of Maryland, Baltimore, USA

The use of medical cannabis (including individual cannabinoids) to treat chronic pain is increasing globally, although such use is not approved by any national regulatory authority such as the US Food and Drug Administration and is not supported by replicated, high-quality controlled clinical trials. The absence of data generated by the standard drug development process leaves clinicians with a substantial knowledge gap regarding appropriate dosing regimens. The article by Bhaskar et al. (2021) attempts to fill this gap by presenting consensus recommendations from an international group of 20 clinicians with extensive experience in recommending medical cannabis as a treatment for chronic pain. The accompanying invited commentary by Hill and Abrams (2021) puts these recommendations into the broader context of the current evidence and lack of evidence regarding medical cannabis as a treatment for chronic pain. Drs. Hill and Abrams are recognized experts in the use of medical cannabis to treat chronic pain. Their commentary and disclosure of competing interests were reviewed and approved by me as Editor-in-Chief; the commentary did not receive external peer review.
